# Malondialdehyde on postoperative day 1 predicts postoperative cognitive dysfunction in elderly patients after hip fracture surgery

**DOI:** 10.1042/BSR20190166

**Published:** 2019-06-20

**Authors:** Chunxian Wu, Bin Gao, Yu Gui

**Affiliations:** Department of Anesthesiology, The Affiliated Hospital of Medical School, Ningbo University, No. 247 Renmin road, Ningbo City 315000, Zhejiang Province, China

**Keywords:** Postoperative cognitive dysfunction, hip fracture, malondialdehyde, oxidative stress, biomarker

## Abstract

**Background:** Postoperative cognitive dysfunction (POCD) is a great problem for anesthetized subjects and is associated with poor short- and long-term outcomes. We explored promising predictors for POCD in elderly patients after hip fracture surgery.

**Methods:** Elderly subjects (aged ≥65 years) undergoing surgery for hip fracture were consecutively recruited. Neuropsychological assessments were performed 1 day preoperatively (baseline) and 7 days postoperatively, and POCD was defined using the ‘*Z* scores’ method. Clinical data and laboratory tests were compared between patients with and without POCD development. Binary univariate and multivariate logistic regression analyses were conducted for risk factor assessment. Receiver operating characteristic (ROC) curve analysis was performed to investigate the predictive value of malondialdehyde (MDA) on postoperative day 1 (POD1) for POCD.

**Results:** A total of 198 patients were finally enrolled in the analysis and 51 patients exhibited POCD within 7 postoperative days, with an incidence rate of 25.8%. MDA expression on POD1 (OR: 1.12, 95%CI: 1.03–1.23, *P*=0.017) was the only independent risk factor for POCD according to the final multivariate logistic regression analysis. ROC curve analysis indicated that MDA on POD1 was a predictor for POCD, with an area under the curve (AUC) of 0.683 and 95%CI of 0.590–0.775 (*P*<0.001).

**Conclusions:** In conclusion, we demonstrated that MDA on POD1 was an independent risk factor for POCD in elderly subjects undergoing hip fracture surgery.

## Introduction

Postoperative cognitive dysfunction (POCD), a condition that has been poorly recognized and defined for decades, is one of the most common postoperative complications, especially for the elderly [1]. As defined by cognitive impairment following surgery with unclear etiology, POCD is a great problem for anesthetized subjects and an important determinant for poor outcomes both short- and long term [2]. Due to significant improvements in medical technology and therapies, life span has been extended, and quality of life has been improved, and requiring more elective surgery than ever. As reported by previous studies, the incidence of POCD varies from 8.9% to 46.1% [3]. Another study reported that the prevalence of POCD in adult patients undergoing noncardiac surgery ranges from 19% to 41% [4]. The occurrence of POCD is significantly associated with a delay in functional recovery and increases in economic burden and mortality rate [5]. Without an adequate plan to address potential cognitive dysfunction, there is a global health risk of increased cognitive decline. The prognosis can be significantly improved if POCD is recognized early and managed effectively [6]. For elderly subjects after hip fracture surgery, POCD is a frequent complication that is closely associated with prolonged hospital stay and increased morbidity and mortality [7]. Due to a lack of understanding of POCD and its multifactorial causes, specific prediction of POCD and its therapy are quite difficult [8]. As a result, preventing the development of POCD is of great importance.

Recently, oxidative stress-induced neuroinflammation has been widely postulated to be closely associated with synapse dysfunction, and oxidative stress has been speculated to underlie the development of POCD in elderly subjects [9]. Operation is hypothesized to be a major cause of the oxidative response in the CNS [10]. Oxidative stress has been widely considered an important part of the postoperative stress response [11]. A previous study which was conducted in experimental POCD model rats reported that oxidative stress contributes to the etiology of POCD [12]. However, no consensus has been reached about the relationship between oxidative stress and POCD until now. MDA, a bioactive aldehyde generated by free radical-mediated lipid peroxidation, is an oxidative stress marker [13]. Superoxide dismutase (SOD) is an important enzyme that catalyses the dismutation of superoxide radicals into hydrogen peroxide (H_2_O_2_) or oxygen (O_2_) [14]. MDA and SOD are two classical indexes reflecting systemic redox homeostasis.

The present study aimed to investigate whether these biomarkers of redox status could independently predict POCD in elderly subjects undergoing hip fracture surgery.

## Materials and methods

### Patients

This current investigation was approved by the Medical Institutional Ethics Committee of the affiliated hospital of medical school, Ningbo University and Zhejiang province. This prospective single-center observational investigation was performed in the Department of anesthesiology from October 2014 to May 2018. Elderly subjects (aged ≥65 years) undergoing surgery for hip fracture were consecutively recruited. All the participants were required to offer written informed consent. The exclusion criteria were described as follows: (1) with preexisting psychiatric disorder (e.g. dementia, depressive illness, delirium, etc.); (2) with present or prior neurological diseases; (3) with a Mini-Mental State Examination (MMSE) score less than 24 before surgery; (4) with a previous history of neurosurgical surgery; (5) with alcoholism or drug dependence; (6) with auditory or visual disorders, inability to communicate with Chinese language; (7) not willing to participate in the present study; (8) with data missed.

### Neuropsychological assessment and POCD definition

A same experienced anesthetist who was blinded to the present study was invited to perform the neuropsychological assessment on 1 day preoperatively (baseline) and 7 day postoperatively. The neuropsychological assessments comprised the widely described tests including MMSE, Word recognition memory test, Digit span test, Trail making test (part A), Symbol digit test, and Verbal fluency test [15]. A *Z* score for each test was then calculated using the baseline levels and test results according to the ISPOCD1 study by Moller et al. [16]. In this current study, POCD was defined using ‘*Z* scores’ according to the established method in the ISPOCD1 study [16]. If two (or more) *Z* scores in individual tests or the combined *Z* score were ≥1.96, POCD was diagnosed.

### Clinical data collection

The demographic data including age, gender, body mass index (BMI), American Society of Anesthesiologists (ASA) grade, and smoking status were recorded at initial recruitment. We also collected the preoperative comorbidities (diabetes, hyperlipidemia and hypertension) and calculated the MMSE scores before the surgery. In addition, the clinical data associated with the surgery including type of fracture, anesthesia and surgery, delay of surgery, duration of anesthesia and surgery, recovery time, and perioperative blood transfusion rate were also recorded in details.

### Laboratory tests

Fasting blood samples on 1 day before surgery (baseline) and postoperative day 1 (POD1) were collected. The whole blood samples were then centrifuged at 3000 rpm for 10 min and the obtained serum samples were stored at −80°C. MDA level was measured by spectrophotometric method using thiobarbituric acid (TBA) and MDA kits (Jiancheng Bioengineering, Nanjing, Jiangsu, China). The determination of MDA levels relied on the reaction with TBA to generate the products ‘thiobarbituric acid reactive substances’ (TBARS) that could be measured by the method of fluorimetry (excitation at 532 nm and emission at 553 nm) or colorimetry (532 nm). The level of MDA was measured colorimetrically in this current study. SOD activity was measured by the hydroxylamine method using SOD kits (Jiancheng Bioengineering, Nanjing, Jiangsu, China). The determination of SOD activity was based on the suppression role of SOD on the superoxide anion through a dismutation reaction. C-reactive protein (CRP) was measured by the method of enzyme-linked immunosorbent assay (ELISA) using kits (R&D Systems, Minneapolis, MN, U.S.A.). The biochemical determinations were carried out in duplicates in triplicate and the mean value was recorded. Hemoglobin, white blood cell, albumin, urea and creatinine were also measured using the preoperative blood samples in the laboratory of our hospital.

### Statistical analysis

Data analyses were performed using GraphPad Prism 5.0 (GraphPad Inc., CA, U.S.A.) and SPSS 19.0 (SPSS Inc., IA, U.S.A.). Before the study, we performed a sample size estimation and 165 patients would be required with a 5% significance level and 80% power. Our final analysis included a sample size of 198 patients and the power value was calculated to be 0.86. All continuous data were reported as mean with standard error (S.E.M) and categorical data as number (*n*) with percentage (%), respectively. Statistical analyses were investigated using Mann–Whitney *U*-test or *t-*test for continuous data, Chi-square test or Fisher exact test for categorical data as appropriate. Binary univariate and multivariate logistic regression analyses were conducted for risk factors assessment. Receiver operating characteristic (ROC) curve analysis was performed to investigate the predictive value of MDA on POD1 for POCD and the cut-off value. A two-sided *P*-value < 0.05 was considered significantly different.

## Results

### Patient characteristics

During the inclusion period, we enrolled 224 elderly patients undergoing hip fracture surgery. Twenty-six were excluded due to the exclusion criteria (five preoperative MMSE score <24, three with preexisting psychiatric disorder, three with a history of neurosurgical surgery, four with auditory or visual disorders, five not willing to cooperate, and six with data missed) and a total of 198 patients were finally enrolled into the analysis. Ultimately, 51 patients have exhibited POCD within 7 postoperative days, with an incidence rate of 25.8%. The detailed clinical characteristics are presented in Table 1. The age and ASA grade were significantly higher in the POCD group (*P*=0.045 and *P*=0.034, respectively). The development of POCD also seemed to be closely associated with a lower preoperative MMSE score (*P*=0.034). The presence of preoperative comorbidities including diabetes (*P*=0.036) and hypertension (*P*=0.027) was significantly associated with an increased risk of POCD. Those patients who underwent surgery under general anesthesia (*P*=0.010) or those who had a longer duration of surgery (*P*=0.042) or anesthesia (*P*=0.026) were more likely to suffer POCD. No significant differences were observed in gender distribution, smoking status, type of fracture and surgery, delay of surgery, recovery time, perioperative blood transfusion, and baseline neuropsychological assessments (including Digit span test, Verbal fluency test, etc.) between the patients with or without POCD (*P*>0.05).

**Table 1:** Demographic and clinical data and POCD

Variables	POCD (*n*=51)	Non-POCD (*n*=147)	*P*-value
Age (year)	72.3 ± 3.2	71.3 ± 3.0	0.045*
Gender, *n* (%)			0.71
Male	22 (43.1)	59 (38.1)	–
Female	29 (56.9)	88 (61.9)	–
BMI (kg/m^2^)	23.2 ± 2.1	22.9 ± 1.9	0.35
ASA physical status, *n* (%)			0.034*
I	3 (5.9)	18 (12.2)	–
II	20 (39.2)	78 (53.1)	–
III	28 (54.9)	51 (34.7)	–
Active smoker, *n* (%)	10 (19.6)	24 (16.3)	0.59
Preoperative comorbidities, *n* (%)			–
Diabetes	13 (25.5)	19 (12.9)	0.036*
Hyperlipidemia	9 (17.6)	25 (17.0)	0.92
Hypertension	17 (33.3)	27 (18.4)	0.027*
Preoperative medications, *n* (%)			
Hypoglycemic drugs	10 (19.6)	16 (10.9)	0.11
Beta blocker	6 (11.8)	11 (7.5)	0.35
ACEI/ARB	9 (15.7)	16 (10.9)	0.21
Calcium channel blocker	6 (11.8)	9 (6.1)	0.35
Lipid-lowering medication	6 (11.8)	15 (10.2)	0.76
Type of fracture			0.85
Femoral neck fracture	28 (54.9)	83 (56.5)	–
Intertrochanteric fracture	23 (45.1)	64 (43.5)	–
Type of anesthesia			0.010*
Spinal	21 (41.2)	91 (61.9)	–
General	30 (58.8)	56 (38.1)	–
Type of surgery			0.28
Arthroplasty	41 (80.4)	107 (72.8)	–
Internal fixation	10 (19.6)	40 (27.2)	–
Delay of surgery (days)	3.7 ± 1.5	3.5 ± 1.8	0.48
Duration of surgery (min)	98.3 ± 16.2	93.5 ± 13.8	0.042*
Duration of anesthesia (min)	110.5 ± 18.1	104.8 ± 14.8	0.026*
Recovery time (min)	35.5 ± 8.8	36.1 ± 10.1	0.71
Perioperative blood transfusion, *n* (%)	14 (27.5)	33 (22.4)	0.47
Preoperative neuropsychological assessments			
MMSE score	27.5 ± 1.5	28.1 ± 1.8	0.034*
Digit span test			
Correct order	8.4 ± 0.7	8.3 ± 0.8	0.43
Reverse order	4.5 ± 1.3	4.4 ± 1.1	0.59
Trail making test A (s)	19.1 ± 8.7	18.2 ± 6.9	0.46
Verbal fluency test	15.2 ± 3.5	16.2 ± 3.9	0.11
Word recognition memory tests	1.4 ± 1.0	1.3 ± 0.9	0.51
Symbol digit test	31.5 ± 10.8	32.4 ± 9.7	0.50

*P<0.05.

### Laboratory characteristics and POCD

The laboratory tests in patients with or without POCD are listed in Table 2. The content of hemoglobin, white blood cell, albumin, creatinine, and urea were not statistically significant (*P*>0.05). The preoperative levels of CRP, MDA, and SOD in patients with POCD also did not differ from those without POCD (*P*>0.05). Those patients with POCD exhibition showed significantly increased serum levels of CRP and MDA and decreased activity of SOD on POD1 (*P*<0.05).

**Table 2:** The laboratory tests and POCD

Variables	POCD (*n*=51)	Non-POCD (*n*=147)	*P*-value
Hemoglobin (g/dl)	11.1 ± 1.9	11.5 ± 1.7	0.16
White blood cell (×10^9^/L)	7.6 ± 1.9	7.4 ± 1.7	0.48
Albumin (g/ml)	39.7 ± 3.8	39.5 ± 3.3	0.72
Creatinine (mmol/L)	82.1 ± 13.3	81.6 ± 14.1	0.82
Urea (mmol/L)	6.4 ± 1.8	6.2 ± 1.5	0.43
Preoperative CRP (mg/L)	11.2 ± 3.6	10.9 ± 4.1	0.64
Preoperative MDA (nmol/ml)	4.5 ± 0.5	4.4 ± 0.6	0.28
Preoperative SOD (U/ml)	94.7 ± 2.9	95.0 ± 2.8	0.51
CRP on POD1 (mg/L)	55.7 ± 22.5	47.4 ± 19.3	0.012*
MDA on POD1 (nmol/ml)	5.9 ± 0.9	5.4 ± 0.7	0.001*
SOD on POD1 (U/ml)	78.7 ± 3.9	80.5 ± 4.3	0.009*

*P<0.05.

### Risk factors for POCD

As mentioned above, all the related risk factors (*P*<0.05 in Tables 1 and 2) were included in the univariate logistic regression analysis. As illustrated in Table 3, age, type of anesthesia, CRP, MDA, and SOD on POD1 were five potential risk factors for POCD (*P*<0.05). Of these five factors, the MDA level on POD1 (OR: 1.12, 95%CI: 1.03–1.23, *P*=0.017) was the only independent predictor for POCD according to the final multivariate logistic regression analysis.

**Table 3:** Univariate and multiple logistic regression analyses for POCD

Variables	Univariate		Multivariate	
	OR(95%CI)	*P-*value	OR(95%CI)	*P-*value
Age	2.23(1.07–4.63)	0.022*	1.44(0.29–2.51)	0.57
ASA grade (I/II vs III)	1.23(0.64–2.36)	0.58		
Diabetes	1.05(0.95–1.17)	0.24		
Hypertension	2.24(0.46–5.37)	0.28		
Preoperative MMSE	0.96(0.75–1.24)	0.57		
Type of anesthesia (spinal vs general)	2.58(1.29–5.11)	0.009*	1.48(0.93–2.38)	0.17
Duration of surgery	1.09(0.54–2.13)	0.76		
Duration of anesthesia	1.69(0.81–3.53)	0.17		
CRP on POD1	2.08(1.07–4.01)	0.033*	1.38(0.72–2.91)	0.13
MDA on POD1	1.14 (1.06–1.24)	0.002*	1.12(1.03–1.23)	0.017*
SOD on POD1	1.59 (1.17–2.18)	0.015*	1.56(0.84–2.87)	0.15

CI, confidence interval; OR, odds ratio. *P<0.05.

### MDA on POD1 and POCD

**Figure 1:**
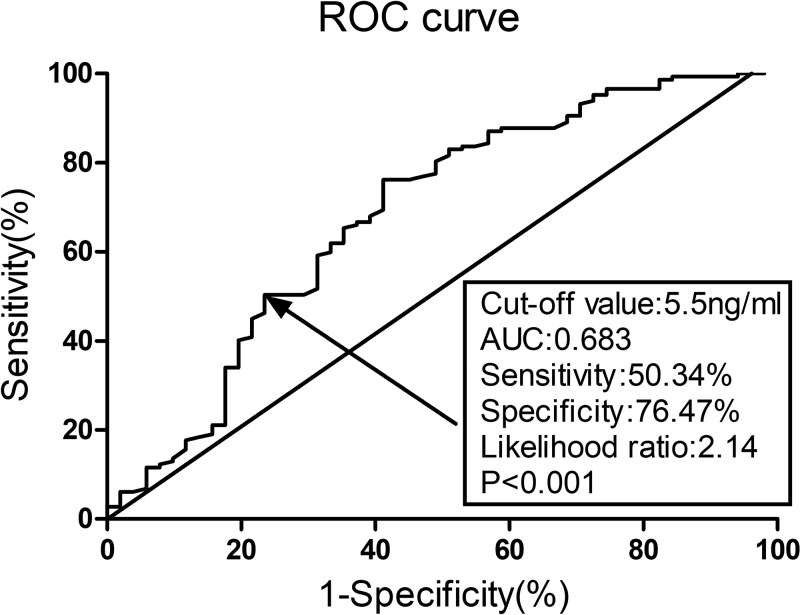
Predictive value of MDA on POD1 for POCD by ROC curve analysis MDA on POD1 was a predictor for POCD, with an AUC of 0.683, 95%CI of 0.590–0.775, a cut-off value of 5.5 nmol/ml, a sensitivity of 50.34%, and a specificity of 76.47%, respectively (*P*<0.001). POD, postoperative day; CI, confidence interval.

As shown in Figure 1, ROC curve analysis indicated that the MDA level on POD1 was a predictor for POCD, with an area under the curve (AUC) of 0.683, 95%CI of 0.590–0.775, a cut-off value of 5.5 nmol/ml, a sensitivity of 50.34%, and a specificity of 76.47% (*P*<0.001).

## Discussion

In the present study, MDA expression on POD1 was an independent risk factor for POCD in elderly subjects undergoing surgery for hip fracture. The incidence rate of POCD within 7 postoperative days in our investigation was 25.8%, which was quite similar to the 29.9% reported by Ji et al. [17], 29.2% by Papadopoulos et al. [18], and 26.1% by Cheng et al. [19].

POCD is a neurological complication after surgery, and is very common in the elderly surgical patients [20]. POCD may lead to obstacles of visual memory, verbal memory, visuospatial abstraction, language comprehension, concentration, or attention [21]. To our knowledge, our understanding of the pathophysiology of POCD is relatively limited [22]. Researchers have performed numerous studies to investigate risk factors for POCD. Advanced age is universally identified as an important risk factor for POCD, indicating a basic role of brain tissue changes in POCD [23]. An increasing age is closely associated with an elevated incidence of endothelial dysfunction and cerebral atherosclerosis, which may lead to POCD [24]. However, our multivariate analysis did not support age as an independent risk factor for POCD. We considered that the relatively small range of age in the present study is the main explanation for this finding. The pathophysiology of POCD is generally agreed to be multifactorial, and whether POCD development is caused by general anesthesia remains unclear [25]. The traditional view holds that anesthesia is associated with an increased incidence of POCD. However, there were some studies that argued to the contrary [26]. A review by Rundshagen has indicated that general anesthesia does not play a causal role in POCD development [27]. A qualitative systematic review by Paredes et al. also indicated that general anesthesia is not significantly associated with the occurrence of POCD [28], which is in agreement with our results. It is well known that inflammatory response plays a critical role in activation of glial cell that leads to brain injury during POCD [29]. Accumulating evidence has disclosed that high levels of CRP are closely correlated with increased risks of POCD or postoperative delirium (POD) [30]. Some studies have revealed a significant association between serum CRP concentrations and POCD, indicating a critical role of the inflammatory response in POCD development [31,32]. However, some other researchers found that CRP was not an independent biomarker of cognitive impairment [33]. Indeed, our results did not support a predictive role of CRP for POCD.

The oxidative stress is associated increased proapoptotic proteins and decreased anti-apoptotic proteins in hippocampus and frontal cortex [34]. Furthermore, Zhang et al. has reported that neuronal apoptosis contributes to POCD, indicating the strong correlations between oxidative stress and POCD [35]. SOD is a vital enzyme that catalyzes the conversion of the superoxide radicals into hydrogen peroxide (H_2_O_2_) or oxygen (O_2_) [14]. The impairment of SOD has been reported to link with oxidative stress [36]. Some studies have reported that SOD is linked to neurocognitive deficits [37]. Alteration in SOD has been linked to the cellular apoptotic signaling introduced by mitochondrial factors or transcriptional factors [36]. However, no studies, including this present study, have reported the predictive role of SOD for POCD. MDA is a significant indicator of oxidative stress and can directly reflect plasma membrane damage induced by free radicals [38]. The overproduction of MDA is stimulated by increased free radicals [39], and MDA is used as an important biomarker for lipid peroxidation and peroxidative tissue injury [40]. MDA is widely used as a reliable and popular biomarker for oxidative stress assessment in clinical applications [41]. Our results indicated that MDA on POD1 was an independent risk factor for POCD, strongly suggesting a close association between oxidative stress and POCD. Excessive oxidative stress leads to lipid peroxidation of the cell membranes and the subsequent accumulation of MDA can damage brain cells, which may result in cognitive decline [13]. Pan et al. demonstrated that robustly increased hippocampal MDA after surgery was significantly associated with changes in postoperative cognition [42]. In addition to direct injury on brain cells, the complicated cross-talk between oxidative stress and the innate immune cells in the central nervous system is also a vital issue for the development of cognitive decline [42]. These points might at least partly explain the increased MDA levels in patients with POCD. A study conducted in an experimental model of tibial fracture in rats demonstrated that oxidative stress and mitochondrial dysfunction are two important contributors for POCD [12]. Notably, mitochondrial function can be impaired by oxidative stress via inducing structural changes [43]. Accumulated evidence has revealed that oxidative stress contributes to varied neurodegenerative disorders [44]. The vicious circle of a sustained oxidative environment and neuroinflammation in response to oxidative stress can damage healthy nerve cells, resulting in synapse dysfunction [45]. All these reports have indicated a close correlation between oxidative stress and POCD, which offers a possible explanation for the predictive role of MDA in POCD.

However, the present study has some great limitations. First, the inclusion of patients with oxidative stress-related diseases (e.g., diabetes and hypertension) and the medicines taken by the patients taken are factors that may have greatly influenced the results. Second, the determination of MDA by colorimetry or fluorimetry has historically relied on a reaction with TBA. However, this method of MDA measurement lacks sensitivity and specificity due to the possible reaction of TBA with some chemically reactive carbonyl group containing compounds (sugars, amino acids, etc.). Therefore, MDA may not be an ideal marker of lipid peroxidation as expected. Finally, only two biomarkers were measured for oxidative stress evaluation and they do not fully explain the redox homeostasis in POCD pathology.

## Conclusions

In conclusion, we demonstrated that MDA on POD1 was an independent risk factor for POCD in elderly subjects undergoing hip fracture surgery. Furthermore, we suggest that oxidative stress intervention may be a promising strategy for POCD prevention.

## Abbreviations

ASA: American Society of Anesthesiologists
AUC: area under the curve
BMI: body mass index
CRP: C-reactive protein
MDA: malondialdehyde
MMSE: Mini-Mental State Examination
POCD: postoperative cognitive dysfunction
POD1: postoperative day 1
ROC: receiver operating characteristic
SOD: superoxide dismutase
TBA: thiobarbituric acid
